# Association Between Serum Magnesium Levels and Suicidal Behavior in Patients Diagnosed With Depression: A Cross-Sectional Case-Control Study

**DOI:** 10.7759/cureus.91656

**Published:** 2025-09-05

**Authors:** Kapil Raghuwanshi, Abhay Kumar, Anjulika Mehta, Mahendra Gandhe, Bharti Badlani, Keerthi Sanapala

**Affiliations:** 1 Biochemistry, Chhindwara Institute of Medical Sciences, Chhindwara, IND; 2 Otolaryngology, Chhindwara Institute of Medical Sciences, Chhindwara, IND; 3 Physiology, Chhindwara Institute of Medical Sciences, Chhindwara, IND; 4 Ophthalmology, Chhindwara Institute of Medical Sciences, Chhindwara, IND; 5 Internal Medicine, Andhra Medical College, Visakhapatnam, IND

**Keywords:** depression, mental health, neurotransmitter dysregulation, serum magnesium, suicide

## Abstract

Background: Depression is a common psychiatric illness characterized by persistent low mood, anhedonia, and reduced energy. Severe forms of depression are associated with increased risk of suicidal behavior. Emerging evidence suggests that micronutrient imbalances, including deficiencies in magnesium, vitamin D, zinc, and B-complex vitamins, may influence neuropsychiatric functioning by affecting neurotransmitter synthesis, neuroinflammation, and synaptic plasticity, thereby impacting mood regulation.

Methodology: This cross-sectional observational study was conducted at a government medical college in Madhya Pradesh. A total of 200 patients attending the psychiatry outpatient department were enrolled: 100 diagnosed with major depressive disorder and suicidal behavior (case group), and 100 age- and sex-matched patients with depression but without suicidal behavior (control group). Depression severity was assessed using the Hamilton Rating Scale for Depression (HAM-D), and suicidal behavior was evaluated using the Revised Suicidal Behavior Questionnaire (SBQ-R). Ethical approval and informed consent were obtained prior to participation.

Results: The mean serum magnesium level was significantly lower in the case group (1.59 ± 0.19 mg/dL) compared to the control group (1.90 ± 0.10 mg/dL, p < 0.001). In both groups, magnesium levels decreased as the severity of depression (measured by the HAM-D scale) increased. A statistically significant negative correlation was found between serum magnesium levels and suicidal behavior scores (SBQ-R) in the case group (r=-0.612, p<0.001).

Conclusion: The findings of this study indicate that lower blood magnesium levels are associated with more severe depression in both study groups. Measuring serum magnesium levels at the time of diagnosis may aid in providing more personalized treatment plans. Additionally, magnesium supplementation could be explored further as an adjunct strategy to reduce depressive symptom severity and suicidal behavior risk.

## Introduction

Depression is a psychiatric disorder characterized by persistent low mood, anhedonia, and decreased energy lasting at least two weeks. Globally, depression affects approximately 3.4% of the population, with a higher prevalence in women than in men. Furthermore, 16.2% of people experience depression during their lifetime [1]. Depression is caused by a combination of biological, psychological, psychosocial, and developmental factors. The monoamine hypothesis, which contends that depression is caused by either a lack of the neurotransmitter norepinephrine and/or serotonin or by a loss of sensitivity of the appropriate receptors, is one theory of how depression begins [2]. Micronutrients are essential for neurotransmitter synthesis and nervous system function, and deficiencies have been linked to mental health disorders. Therefore, insufficient consumption of micronutrients negatively impacts psychological functioning and may increase the risk of depressive illnesses. According to Li et al. [3], a higher incidence of depression is linked to a lower dietary intake of magnesium.

Deficiencies in these micronutrients, particularly magnesium, may also be associated with increased risk of suicidal ideation and behavior, highlighting the importance of studying magnesium status in depressive patients. Magnesium supports neuromuscular conduction, neurotransmission, calcium homeostasis, and neuroinflammatory regulation. It also modulates NMDA receptor activity and protects against excitotoxicity and neuronal damage. It also regulates mood, neuroinflammation, and synaptic plasticity [4]. At low levels, glutamatergic signaling may become overactive, contributing to oxidative stress and neuronal damage [5]. Molecular studies suggest magnesium may have neuroprotective effects in humans. Research has shown that emotional disorders and electrolyte imbalances are closely associated. Severe depression can lead to suicidal ideation and behavior [6]. Suicidal behavior encompasses a range of actions, including suicidal thoughts, threats, attempts, and actual suicide. Many individuals with depression do not receive help until they have attempted suicide. A prior suicide attempt is a reliable indicator of subsequent attempts [7]. Suicide can be avoided, but it requires concerted efforts from friends, family, and medical experts.

However, few studies have examined the relationship between electrolyte abnormalities and suicidality in patients with depression. For example, Li et al. [3] found an association between lower dietary magnesium intake and higher risk of depression in the general population, but comprehensive studies specifically evaluating serum magnesium levels and suicidality in depressive patients are scarce. To test the hypothesis that serum magnesium levels in suicidal patients would be lower than those in non-suicidal controls, our study aimed to assess the serum magnesium levels in patients with depression. Given these physiological roles, reduced magnesium levels may influence mood regulation and contribute to the pathophysiology of depressive and suicidal states.

Objectives

This study aimed to evaluate serum magnesium levels in patients diagnosed with depression, comparing those with suicidal behavior to those without. Additionally, it sought to determine whether suicidal behavior is related to serum magnesium levels and to assess whether magnesium levels decrease with increasing depression severity. These objectives were designed to clarify the potential relationship between magnesium status and both depression severity and suicidal tendencies.

## Materials and methods

Study design

This study employed a cross-sectional case-control design to evaluate the association, not temporal or causal relationships, between serum magnesium levels and suicidal behavior among patients diagnosed with depression. This study was conducted in the Department of Psychiatry and Biochemistry of the Government Medical College, Chhindwara Institute of Medical Sciences (C.I.M.S.), Chhindwara, Madhya Pradesh, India. As a single-center, hospital-based study, the findings may primarily reflect urban outpatient populations.

Study population

A total of 200 consecutive eligible patients, aged 18 to 50 years, were enrolled in the psychiatry outpatient department of the District Hospital, Chhindwara, affiliated with Chhindwara Institute of Medical Sciences (C.I.M.S.), Chhindwara, Madhya Pradesh, India. The case group consisted of 100 patients with a diagnosis of depression and suicidal behavior (either suicidal thoughts or suicide attempts), whereas the control group consisted of 100 patients with a diagnosis of depression who were matched for age and sex but did not exhibit suicidal behavior.

Sample size

To enable comparisons between groups, the sample size of 200 (100 per group) was determined based on feasibility and prior literature suggesting that this would be sufficient to detect meaningful differences in serum magnesium levels between groups (alpha = 0.05, power = 80%). The sample size was deemed sufficient to ensure data collection feasibility and compliance with time and resource constraints, as well as to detect statistically significant differences in serum magnesium levels between the groups. Age- and sex-matching were used to control for confounding variables.

Inclusion criteria

This study included participants with a diagnosis of depression, having suicidal behavior, including attempts or thoughts (for the case group), not having suicidal behavior (for the control group), being between the ages of 18 and 50, being either male or female, drug-naïve or drug-free for three months, and being prepared to provide written informed consent.

Exclusion criteria

Patients who were taking antidepressant medications, were pregnant or nursing, had an active infection, were dependent on alcohol or tobacco, had any autoimmune inflammatory disease, had intellectual disability, had serious medical conditions (such as cancer or bedridden patients), or were unwilling to provide their consent were excluded.

Data collection procedures

Eligible participants were enrolled successively in the case and control groups, following the acquisition of written informed consent. A structured data collection form was used to record clinical and demographic data such as age, sex, length of illness, and current symptomatology. The Revised Suicidal Behavior Questionnaire (SBQ-R) was used to measure suicidal behavior, and the 17-item Hamilton Rating Scale for Depression (HAM-D) was used to gauge depression severity. All assessments were conducted by trained clinicians who were blinded to group allocation to minimize bias. Venous blood samples were collected from each participant under aseptic conditions. Serum magnesium levels were measured using spectrophotometric techniques on a Biosystem BA400 fully automated biochemist analyzer after the samples were centrifuged to separate the serum. 

Quality control 

All biochemical analyses were performed in a carefully monitored laboratory in the Department of Biochemistry. To guarantee the consistency and accuracy of serum magnesium estimation, the apparatus was calibrated on a regular basis, and internal quality control (IQC) checks were performed using commercial control sera before analysis. To reduce errors, independent staff double-checked the data entry, and only validated entries were included in the final analysis. To reduce subjective bias, qualified personnel who followed established protocols administered the Hamilton Rating Scale for Depression (HAM-D) [8] and the Suicidal Behaviors Questionnaire-Revised (SBQ-R) [9]. The HAM-D is in the public domain; however, the SBQ-R is a copyrighted instrument, and its use in this research was done in accordance with the terms of the copyright holders. Selection bias was minimized by consecutively enrolling all eligible participants in the study. Assessment bias was reduced by ensuring that psychometric evaluations were conducted by raters who were blinded to group allocation. Age and sex confounding factors were controlled by matching. There were no missing data for the primary variables; all records were complete and included in the final analyses.

Descriptive analysis

The clinical and demographic variables were compiled using descriptive statistics. The mean ± standard deviation (SD) was used to express continuous variables, such as age, serum magnesium levels, HAM-D score, and SBQ-R score. Frequencies and percentages were used to display categorical variables such as the distribution of sex and severity categories of depression. Microsoft Excel was used to organize the data, and IBM SPSS version 15.0 was used for analysis.

Inferential statistics

An independent sample t-test was used to compare the serum magnesium levels between the case and control groups. One-way analysis of variance (ANOVA) was used to compare the mean magnesium levels among the depression severity groups. The association between serum magnesium levels and the HAM-D or SBQ-R scores was evaluated using Pearson's correlation coefficient. Statistical significance was defined as a p-value of less than 0.05.

Ethical considerations

After receiving approval from the Institutional Ethics Committee, this cross-sectional case-control study was conducted at the Chhindwara Institute of Medical Sciences (C.I.M.S.) Chhindwara, Madhya Pradesh, India (CIMS/Ethics Committee/2022/6388, Date: 31/08/2022).

Informed consent and voluntary participation

All participants received information regarding the study's goals, methods, possible advantages, and risks. Prior to inclusion in the study, each participant provided a written informed consent. The participants were made aware of the freedom to leave the study at any time without affecting their medical treatment, and participation was completely voluntary. By anonymizing data and securely storing responses, all personal and medical information was kept strictly confidential throughout the study.

## Results

Demographic and clinical characteristics

Our research revealed several important findings: patients with depression who engaged in suicidal behavior had an average age of 34.91 ± 5.51 years, whereas those who did not had an average age of 35.76 ± 5.42 years. There were no statistically significant differences between the groups in terms of age (p = 0.27) and length of illness (p = 0.81), confirming appropriate matching. The average length of illness was 7.17 ± 1.29 months for patients with suicidal conduct and 7.12 ± 1.71 months for those without suicidal behavior. The mean HAM-D score was significantly higher in the case group (17.5 ± 4.10) than in the control group (15.62 ± 3.20) (p < 0.001), indicating greater depression severity among patients with suicidal behavior. According to the HAM-D scale interpretation, these mean scores correspond to moderate depression severity in both groups, with higher severity in the case group. Patients with depression exhibiting suicidal conduct had an average score of 15.16 ± 2.92 on the SBQ-R. 

Serum magnesium levels: case vs. control groups

Patients with depression and suicidal behavior had a mean serum magnesium level of 1.59 ± 0.19 mg/dl, whereas those without suicidal behavior had a mean serum magnesium level of 1.90 ± 0.10 mg/dl (Table 1).

**Table 1:** Comparison of serum magnesium between case and control groups Independent sample t-test; statistically significant (p < 0.001)

Parameter	Group	Count	Mean ± SD	t-test	p-value
Serum Magnesium (mg/dl)	Case group	100	1.59 ± 0.19	14.19	< 0.001
	Control group	100	1.90 ± 0.10		

Serum magnesium levels and depression severity: case vs control groups

Patients with depression and suicidal behavior had significantly lower blood magnesium levels than those without such behavior. There was a significant difference in blood magnesium levels between patients with and without suicidal behavior, depending on the severity of depression. This finding showed that blood magnesium levels decreased with increasing depression in both groups (Tables 2, 3).

**Table 2:** Comparison of mean serum magnesium in different severity groups of depression in the case group One-way ANOVA; p < 0.001: highly significant; p < 0.05: significant

Parameter	Case group	Count	Mean ± SD	f-value	p-value
Serum magnesium (mg/dl)	Mild depression	21	1.85 ± 0.04	135.11	< 0.001
	Moderate depression	22	1.71 ± 0.07		
	Severe depression	57	1.45 ± 0.12		

**Table 3:** Comparison of mean serum magnesium in different severity groups of depression in the control group One-way ANOVA; p < 0.001: highly significant; p < 0.05: significant

Parameter	Control group	Count	Mean± SD	f-value	p-value
Serum magnesium (mg/dl)	Mild depression	23	2.01 ± 0.08	62.28	< 0.001
	Moderate depression	66	1.90 ± 0.06		
	Severe depression	11	1.73 ± 0.06		

Serum magnesium levels and HAM-D score correlation: case vs. control groups

In individuals with depression who exhibited suicidal behavior, there was a moderately negative and statistically significant association between serum magnesium levels and SBQ-R (-0.612) scores (Table 4).

**Table 4:** Correlation of serum magnesium eith HAM-D and SBQ-R scores in depressed patients Pearson’s correlation coefficient; p < 0.001: highly significant; p < 0.05: significant HAM-D: Hamilton Rating Scale for Depression; SBQ-R: Revised Suicidal Behavior Questionnaire

Scale	Group	r	p-value	Interpretation
HAM-D	Case group	-0.78	< 0.001	Strong negative correlation
HAM-D	Control group	-0.62	< 0.001	Moderate negative correlation
SBQ-R	Case group only	-0.61	< 0.001	Moderate negative correlation

## Discussion

This study demonstrated that serum magnesium levels were significantly and negatively correlated with HAM-D scores in both suicidal and non-suicidal depressed patients. In patients with depression who exhibited suicidal behavior, a moderately unfavorable connection was also observed between serum magnesium levels and SBQ-R scores. 

Numerous studies have reported conflicting findings regarding the serum magnesium levels in patients with depression. In our study, the mean serum magnesium level in the depressed suicidal group was 1.59 ± 0.19 mg/dL and the mean serum magnesium level in the depressed non-suicidal group was 1.90 ± 0.10 mg/dL (p < 0.00001). Jung et al. [10] reported that women from the lowest serum magnesium tertile had 3.92 times higher odds of having a depressive mood disorder (OR = 3.92, 95% CI 1.11-13.83), which support this result. Camardese et al. [11] reported no obvious relationship between magnesium and the initial level of depression, and the majority of patients with depression in their study had normal magnesium levels. In contrast, our results showed that lower magnesium correlated with higher HAM-D scores (r = -0.780) in suicidal patients: responders to antidepressant treatment tended to have greater magnesium than non-responders, supporting the idea that magnesium status could affect treatment response. However, other studies, such as those by Camardese et al. [11], did not find a consistent relationship, highlighting heterogeneity in the literature based on cohort characteristics, rating scales, or analytical differences. Rechenberg et al. [12] reported that people with depression have lower amounts of magnesium in their bloodstream and cerebral fluid than controls.

In a case-control study, Barragán-Rodríguez et al. [13], reported an adjusted association of depression and hypomagnesemia with an OR of 1.79 (95% CI 1.1-6.9) in the diabetic elderly where serum magnesium levels of depressed diabetic elderly were 0.74 ± 0.25 mmol/L versus controls 0.86 ± 0.29 mmol/L (p = 0.02). There was also a clear statistical gradient for severity and suicidal behavior with hypomagnesemia, which was of a similar magnitude and direction, but unlike our results. However, our findings indicated that lower magnesium levels were associated with higher depression scores, while Hasey et al. [14] found a positive correlation between serum magnesium levels and Beck Depression Inventory scores. This discrepancy may be explained by differences in sample sizes, populations, definitions of depression and suicidality, or different depression rating scales. The literature on magnesium and depression shows varied results, with some studies (e.g., Derom et al. [15]) finding no difference, while others report inverse or even positive correlations, highlighting the complexity of this association and the need for further research. These discrepancies across studies may arise from differences in population demographics, methods of magnesium measurement (serum vs intracellular), depression severity definitions, and instruments used to assess depression and suicidality. Such methodological and clinical variations underscore the complexity of interpreting magnesium’s role in depression.

Strengths

The simultaneous clinical psychiatric assessment and biochemical evaluation is a valuable strength of this study, since it allows for a multifaceted examination of the interplay between serum magnesium concentrations and suicidal behavior in depression. A high level of data reliability was ensured because of its methodological monitoring and structure, particularly regarding the quality control for the determination of serum magnesium levels. Trained personnel also constrained subjective bias in data collection to consistently administer validated psychometric instruments in a standardized manner. The usability of these associations has increased because of this methodological rigor and provides a strong rationale for generating hypotheses for future studies.

Limitations

This study had some limitations, although a deliberate methodological approach was used to address them. The interpretation of the findings is limited because of the lack of intracellular or cerebrospinal magnesium measurements, as serum levels alone might not be sufficient to characterize a neurologically relevant magnesium status. As this was a single-center, hospital-based study, the generalizability of the findings to other populations or community-based samples is limited. Additionally, some residual confounding was possible because we did not have data on potential dietary factors, psychosocial stressors, or comorbid nutritional deficiencies that could have influenced serum magnesium levels. Another consideration is the exclusion of antidepressant use. Although this was necessary for biological rigor, it limited the generalizability of the findings to a larger clinical population that used medication. Furthermore, we did not account for potential confounding factors such as dietary magnesium intake, physical activity, or comorbid conditions (e.g., diabetes, renal dysfunction), which could significantly impact serum magnesium levels and should be controlled in future studies.

Implications

These findings have broad implications for research and clinical applications. The association between low serum magnesium levels and suicidal behavior raises important questions regarding the biological vulnerability factors involved in the risk of psychiatric illness. Causality cannot be established from this study; however, future research is necessary to include magnesium status as an additional clinical indicator of suicide risk and to assess micronutrient status in patients with psychiatric disorders. Additionally, the findings indicate a possible pathway for targeted dietary changes that necessitates additional research using prospective interventional designs to examine how mental health outcomes might be influenced by optimizing magnesium.

## Conclusions

On the basis of our results, low serum magnesium concentrations were correlated with the severity of depression and suicidal behavior in patients with major depressive disorder. This correlation suggests that the assessment of magnesium status may be of clinical relevance in the evaluation of depression. We might propose that these results are due to magnesium's effects on neurotransmitter metabolism, transmission of nerve impulses, and synaptic plasticity, but that this putative mechanism needs additional neurobiological confirmation.

Our findings imply that measuring serum levels of magnesium at diagnosis can help in understanding the severity of illness and individualized management. But more studies, such as large-scale and interventional studies, should be done before magnesium supplementation can be advocated as a therapy for depression.

## References

[1] Miller S, Dell'Osso B, Ketter TA (2014). The prevalence and burden of bipolar depression. J Affect Disord.

[2] Rosa-Neto P, Diksic M, Okazawa H (2004). Measurement of brain regional alpha-[11C]methyl-L-tryptophan trapping as a measure of serotonin synthesis in medication-free patients with major depression. Arch Gen Psychiatry.

[3] Li B, Lv J, Wang W, Zhang D (2017). Dietary magnesium and calcium intake and risk of depression in the general population: A meta-analysis. Aust N Z J Psychiatry.

[4] Kirkland AE, Sarlo GL, Holton KF (2018). The Role of Magnesium in Neurological Disorders. Nutrients.

[5] Stroebel D, Casado M, Paoletti P (2018). Triheteromeric NMDA receptors: from structure to synaptic physiology. Curr Opin Physiol.

[6] Clerc P, Young CA, Bordt EA, Grigore AM, Fiskum G, Polster BM (2013). Magnesium sulfate protects against the bioenergetic consequences of chronic glutamate receptor stimulation. PLoS One.

[7] Nock MK, Borges G, Bromet EJ, Cha CB, Kessler RC, Lee S (2008). Suicide and suicidal behavior. Epidemiol Rev.

[8] Hamilton M (1960). A rating scale for depression. J Neurol Neurosurg Psychiatry.

[9] Osman A, Bagge CL, Gutierrez PM, Konick LC, Kopper BA, Barrios FX (2001). The Suicidal Behaviors Questionnaire-Revised (SBQ-R): validation with clinical and nonclinical samples. Assessment.

[10] Jung KI, Ock SM, Chung JH, Song CH (2010). Associations of serum Ca and Mg levels with mental health in adult women without psychiatric disorders. Biol Trace Elem Res.

[11] Camardese G, De Risio L, Pizi G (2012). Plasma magnesium levels and treatment outcome in depressed patients. Nutr Neurosci.

[12] Rechenberg K (2015). Nutritional interventions in clinical depression. Clin Psychol Sci.

[13] Barragan-Rodríguez L, Rodríguez-Morán M, Guerrero-Romero F (2007). Depressive symptoms and hypomagnesemia in older diabetic subjects. Arch Med Res.

[14] Hasey GM, D'Alessandro E, Cooke RG, Warsh JJ (1993). The interface between thyroid activity, magnesium, and depression: a pilot study. Biol Psychiatry.

[15] Derom ML, Sayón-Orea C, Martínez-Ortega JM, Martínez-González MA (2013). Magnesium and depression: a systematic review. Nutr Neurosci.

